# CIP2A protein expression in high-grade, high-stage bladder cancer

**DOI:** 10.1002/cam4.15

**Published:** 2012-07-15

**Authors:** Lisa P Huang, Diana Savoly, Abraham A Sidi, Martin E Adelson, Eli Mordechai, Jason P Trama

**Affiliations:** 1 Oncoveda, Cancer Research Center, Medical Diagnostic Laboratories, L.L.C., A member of Genesis Biotechnology GroupHamilton, NJ, 08690; 2 Department of Urology, The E. Wolfson Medical CenterHolon, Israel; 3 Sackler Faculty of Medicine, Tel-Aviv UniversityTel-Aviv, Israel

**Keywords:** Bladder cancer, cancer biomarker, cancer diagnostic, CIP2A, TCC

## Abstract

Bladder cancer is one of the most common cancers in the United States. Numerous markers have been evaluated for suitability of bladder cancer detection and surveillance. However, few of them are acceptable as a routine tool. Therefore, there exists a continuing need for an assay that detects the presence of bladder cancer in humans. It would be advantageous to develop an assay with a protein that is associated with the development of bladder cancer. We have identified the cancerous inhibitor of PP2A (CIP2A) protein as a novel bladder cancer biomarker. In this study, Western blot analysis was used to assess the expression level of CIP2A protein in bladder cancer cell lines and bladder cancer patient tissues (*n* = 43). Our studies indicated CIP2A protein was abundantly expressed in bladder cancer cell lines but not in nontumor epithelial cell lines. Furthermore, CIP2A was specifically expressed in transitional cell carcinoma (TCC) of the bladder tumor tissues but not in adjacent nontumor bladder tissue. Our data showed that CIP2A protein detection in high-grade TCC tissues had a sensitivity of 65%, which is 3.4-fold higher than that seen in low-grade TCC tissues (19%). The level of CIP2A protein expression increased with the stage of disease (12%, 27%, 67%, and 100% for pTa, pT1, pT2, and pT3 tumor, respectively). In conclusion, our studies suggest that CIP2A protein is specifically expressed in human bladder tumors. CIP2A is preferentially expressed in high-grade and high-stage TCC tumors, which are high-risk and invasive tumors. Our studies reported here support the role of CIP2A in bladder cancer progression and its usefulness for the surveillance of recurrence or progression of human bladder cancer.

## Introduction

Bladder cancer is one of the most common cancers in the United States. In 2011, the National Cancer Institute estimated approximately 70,000 cases of bladder cancer were diagnosed; of those, more than 15,000 will be fetal. Bladder cancer staging is defined as the degree of tumor invasion into the bladder wall [1]. According to the American Cancer Society, the 5-year survival rate for patients diagnosed with bladder cancer is 98% at pT0, 88% at pTa, 63% at pT1, 46% at pT2, and 15% at pT3 [2, 3]. The survival rate for patients with pT_0_ and pTa are very favorable, similar to that of pT1 with low-grade disease. However, 33% of patients at pT1 with high-grade disease will progress to muscle invasive cancer (≥pT2) or incurable metastatic disease. The survival rate for muscle invasive bladder cancer patients is only about 50%. Among the patients with muscle invasive bladder cancer, especially those with high-grade tumors, some will develop metastatic disease even after radical cystectomies. Therefore, identification of novel markers to indicate pathological phenotypes according to histological grade and T stage are considered useful surrogates for assessing the clinical aggressiveness of bladder cancer. These statistics highlight the fact that early detection of bladder cancer is critical for improving prognosis and long-term survival. In North America, the most common type of urothelial tumor diagnosed is transitional cell carcinoma (TCC), which constitutes more than 90% of all bladder cancers [4].

In bladder cancer, the histological grade and tumor stage are both important prognostic parameters. According to the WHO/ISUP standard, there are four types of urothelial tumors – papilloma, papillary urothelial neoplasm of low malignant potential (PUNLMP), low-grade carcinoma, and high-grade carcinoma [5]. The risk of progression increases beyond papilloma, 3% at PUNLMP, 6% at low-grade tumor, and 49% at high-grade tumor. Although most patients with bladder cancer can be treated with organ-sparing therapy, most patients experience either recurrence or progression. Bladder cancer also has the highest recurrence rate of any malignancy. There are great needs for accurate and diligent surveillance, as well as an accurate prognostic assay.

Numerous markers have been tested for bladder cancer detection and surveillance [6]. These markers include complement factor H (BTA-Stat/TRAK), nuclear matrix proteins (NMP22), mucin-like antigens, hyaluronic acid, hyaluronidase, survivin, soluble Fas, telomerase, and detection of chromosomal aneuploidy and deletion using fluorescence in situ hybridization (UroVysion®, Abbott Molecular Inc., Des Plaines, IL). However, none of them are accepted as a routine tool for bladder cancer diagnostics and surveillance due to their associated high-false positivity rates [6]. With an aging US population, there likely exists a growing need for an assay that detects the presence of bladder cancer in humans. Furthermore, the currently available tests have little prognostic value. It would be advantageous to develop a noninvasive assay utilizing a protein that is associated with the development of bladder cancer to assist in evaluating the potential for recurrence or invasive growth of a bladder tumor.

Here, we identified elevated expression of cancerous inhibitor of protein phosphatase 2A (CIP2A) in bladder cancer. In humans, CIP2A is a protein encoded by the KIAA1524 gene, which is located on Chromosome 3 (3q13.13). The CIP2A protein was first identified as a tumor associated antigen in hepatocellular carcinoma and gastric cancer patients by Soo Hoo et al. [7], who also suggested that CIP2A might also serve as an autoantigen in prostate cancer patients. Five years later, Juntilla et al. reported that CIP2A is an oncoprotein that inhibits PP2A and stabilizes c-MYC in human malignancies [8–10]; it was hypothesized that CIP2A binds directly to c-MYC thereby shielding it from PP2A-mediated dephosphorlyation and preventing its degradation. To date, CIP2A has been shown to be overexpressed in several cancers, including gastric cancer, head and neck squamous cell carcinoma, colon cancer, prostate cancer, cervical cancer, and breast cancer [11–15].

The aim of this preclinical exploratory study was to determine whether the expression of CIP2A could distinguish bladder cancer tumor cell lines and tissues from nontumor cell lines and tissues. Thus, based upon a favorable outcome we further examined and report CIP2A protein detection in human bladder cancer tissues.

## Materials and Methods

### Cell lines and subject samples

Bladder cancer cell lines UroTSA, RT-4, T-24, 5637, and TCCSUP; the cervical cancer cell line HeLa; the normal human colon fibroblast cell line CCD112-CoN; and the immortalized normal ectocervical epithelial cell line Ect1 were purchased from American Type Culture Collection (ATCC) (Manassas, VA). T-24 and RT-4 were grown in McCoy's 5A medium supplemented with 10% fetal bovine serum (FBS) (ATCC). TCCSUP and 5637 were grown in RPMI supplemented with 10% FBS. UroTSA, an SV-40 transformed human bladder urothelium cell line, HeLa and 293FT cell lines (Invitrogen, Carlsbad, CA) were maintained in DMEM medium supplemented with 10% FBS. CCD112-CoN cell line was maintained in EMEM medium supplemented with 10% FBS. Ect1 cell line was maintained in keratinocyte-serum free medium supplemented with 0.05 mg/mL bovine pituitary extract, 0.1 ng/mL EGF, and 44 *μ*g/mL CaCl_2_ (Invitrogen). All cell lines were maintained at 37°C in 5% CO_2_.

Bladder tumor tissues and corresponding noncancerous bladder tissues were obtained from consented patients under Israeli Ministry of Health (Protocol no. 902008-0588) and Local Ethics Committee (Protocol no. 1091) approved protocols from Wolfson Medical Center, Holon, Israel (http://ClinicalTrials.gov identifier: NCT00962052). The diagnosis of bladder cancer was made based upon the new World Health Organization (WHO)/International Society of Urologic Pathologists (ISUP) consensus criteria [5]. Patients were classified according to the 1997 American Joint Committee on Cancer (AJCC) staging system [5].

All patients had positive findings on initial diagnosis by cystoscopy. Tissue samples were immediately frozen after removal and stored at −80°C. A section of the tumor tissue was sent for histologic diagnosis to determine the tumor grade and stage. The patients' background and clinical data are summarized in Table 1.

**Table 1:** Bladder cancer patient characteristics

Bladder cancer (TCC)	*N*	Percent
Total number	43	
Age (median)	70	
Gender		
Male	42	97.7
Female	1	2.3
Grade		
Low malignant potential	4	9.3
Low grade	16	37.2
High grade	23	53.5
Stage		
pTa	16	37.2
pT1	11	25.6
pT2	9	20.9
≥pT3	7	16.3

TCC, transitional cell carcinoma.

### CIP2A shRNA knockdown cell line

CIP2A knockdown cell lines were constructed using the Thermo Scientific Open Biosystems Expression Arrest GIPZ Lentiviral shRNAmir system (Cat. no. RHS4430-98912354) according to the manufacturer's instructions (ThermoScientific, Huntsville, AL). To prepare CIP2A-shRNA lentivirus, 5 × 10^5^ 293FT cells were transfected with 10 *μ*g of pGIPZ-CIP2A shRNA and 5 *μ*g of the packaging vectors (pCMVΔR8.2 and pHCMV-G) and grown at 37°C in 5% CO_2_. Supernatants of the transfected cells, containing the lentivirus particles, were collected at 24 and 48 h posttransfection. To obtain the CIP2A knockdown HeLa cell line, HeLa cells were transduced with CIP2A shRNA lentiviral particles and selected using puromycin (2.5 *μ*g/*μ*L) (Sigma-Aldrich, St. Louis, MO).

### Protein lysate preparation

Frozen tissue samples were mechanically homogenized with a pestle in a mortar filled with liquid nitrogen. Cells were suspended and washed with cold PBS before protein preparation. Cells or homogenized tissues were lysed with modified RIPA buffer (250 mM NaCl, 1% NP-40, 0.25% sodium deoxycholate, 50 mM Tris-HCl buffer pH 8.0, 2 mM EDTA, and protease inhibitor cocktail (Calbiochem, San Diego, CA) on ice for 60 min, and were collected by centrifugation at 14,000 × *g* for 20 min at 4°C. The protein concentration was determined using the Bradford method (Bio-Rad, Hercules, CA). The total protein lysate was stored in aliquots at −80°C.

### Western blot

Two CIP2A monoclonal antibodies were purchased from Santa Cruz (Catalog # SC-80662 and Catalog # SC-80660, Santa Cruz, CA). A CIP2A polyclonal antibody was purchased from Bethyl Laboratories (Catalog # A301-454A, Montgomery, TX). All three CIP2A antibodies were tested independently in Western blot analyses, and monoclonal antibody SC-80662 was chosen for the actual experiments described in this report. *β*-actin monoclonal antibodies were purchased from Sigma (Catalog # A2228-200, St. Louis, MO). Briefly, 100 *μ*g total protein was separated by 7.5% SDS-PAGE under reducing conditions and transferred to PVDF membranes. Membranes were blocked with 5% milk in PBST (0.1% Tween-20) for 1 h at room temperature. Membranes were then incubated with (1:1000) diluted primary antibody for 2 h, washed with PBST buffer, and incubated with peroxidase-labeled goat anti-mouse IgG or peroxidase-labeled goat anti-rabbit IgG secondary antibody (1:1000) for 1 h at room temperature (Catalog # HAF007 and HAF008, R&D Systems, Minneapolis, MN). All membranes were visualized using ECL chemiluminescence detection (GE Healthcare, St. Louis, MO) and the GE Gel Imager ImageQuant LAS4000 system.

### Statistical analysis

The frequency association between CIP2A protein expression and pathological status, such as grade and stage of tumor, was analyzed using Fisher's exact probability test. The *P*-value was a result of a two-tailed test. A *P*-value of <0.05 was considered as statistically significant.

## Results

### CIP2A protein expression is increased in bladder cancer cell lines

**Figure 1:**
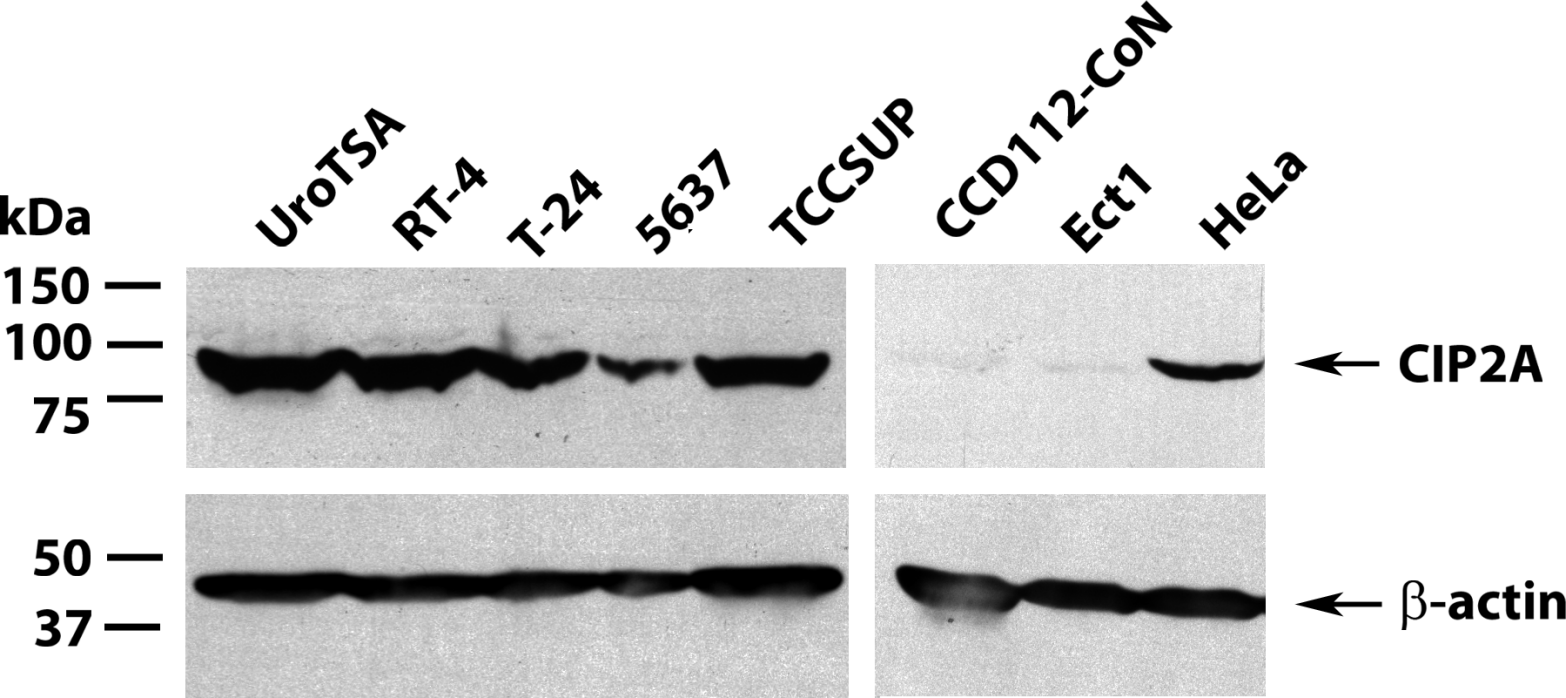
Western blot analysis of CIP2A protein in bladder cells. CIP2A is abundantly expressed in four bladder cancer cell lines (i.e., RT-4, T-24, 5637, and TCCSUP). Note that CIP2A is also expressed in bladder epithelial cells (i.e., UroTSA). No CIP2A expression was found in colon cells (i.e., CCD112-CoN) as well as ectocervical cells (i.e., Ect1). HeLa cervical cancer cell protein lysate was used as positive control for CIP2A protein detection. *β*-actin was used as a loading control.

We examined CIP2A protein expression in various cancer and noncancer cell lines. As shown in Figure 1, all four bladder cancer cell lines expressed CIP2A protein. CIP2A protein was also expressed in the SV40 large T antigen immortalized bladder epithelial cell line, UroTSA. In contrast, CIP2A protein was undetectable in the normal colon fibroblast cell line, CCD112-CoN and the HPV E6/E7 immortalized normal ectocervical cell line, Ect1. Primary human mammary epithelial cells [16] also did not express CIP2A protein (data not shown). We used CIP2A shRNA knockdown HeLa cells to test the specificity of CIP2A monoclonal and polyclonal antibodies. Both monoclonal antibodies SC-80662 and SC-80660, and polyclonal antibody, A301-454A, specifically detected CIP2A protein as a single band (data not shown). These antibodies were all used independently in Western blot analyses to confirm the results in the studies described in this report.

### CIP2A protein is expressed in bladder cancer

In this study, we examined if CIP2A is expressed in bladder cancer tissues. To do so, we obtained snap-frozen bladder tissues from normal adjacent sites (*n* = 43), and tumor sites (*n* = 43) from TCC bladder cancer patients (Table 1). Pathology and histopathological diagnoses were performed at Wolfson Medical Center, Israel. The median age of the patients at the time of diagnosis was 70 years (range 47–86). The majority of patients were male (97.7%) and one patient was female. Patients with low malignant potential, low grade, and high grade were 9.3%, 37.2%, and 53.5%, respectively. Patients with pTa, pT1, pT2, and pT3 or above were 37.2%, 25.6%, 20.9%, and 16.3%, respectively.

**Figure 2:**
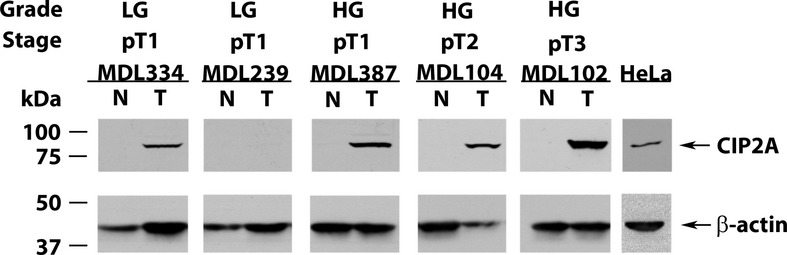
Western blot analysis of CIP2A protein in representative bladder tissues. MDL334: pT1, low-grade TCC; MDL239: pT1, low-grade TCC; MDL387: pT1, high-grade TCC; MDL104: pT2, high-grade TCC; and MDL102: pT3, high-grade TCC. TCC tumor tissues (T) from patients with bladder cancer were examined as well as their adjacent normal tissues (N) from the same patients as a comparison. HeLa cervical cancer cell protein lysate was used as positive control for CIP2A protein detection. *β*-actin served as a loading control. LG, low grade; HG, high grade.

Total cell lysates were prepared from tissues using modified RIPA buffer and analyzed using Western blot assay. CIP2A monoclonal antibody (Santa Cruz) was used for the specific detection of CIP2A protein expression in tissues (Fig. 2). HeLa total cell lysate extracts were used as a control.

Of 43 bladder cancer tissues, CIP2A was expressed in 18 of them. None of the 43 normal adjacent tissues expressed CIP2A protein. This study determined the overall sensitivity and specificity of CIP2A protein expression in bladder cancer tissue to be 42% and 100%, respectively (Table 2).

**Table 2:** CIP2A protein expression in bladder cancer tissue

TCC	CIP2A protein biomarker expression		*P*
Overall	42% (18/43)		
Grade			
Low malignant potential	0% (0/4)		0.008^1^
Low grade	19% (3/16)		
High grade	65% (15/23)		
Stage			
Noninvasive			
pTa	12% (2/16)	19% (5/27)	0.00008^2^
pT1	27% (3/11)		
Invasive			
pT2	67% (6/9)	81% (13/16)	
≥pT3	100% (7/7)		

1 High-grade versus low-grade TCC.

2 Invasive versus noninvasive TCC.

### CIP2A protein expression is elevated in high-risk bladder cancer patients

The percentage of CIP2A protein positive tissue increased with the grade of bladder tumor, 0% in low malignant potential, 19% in low grade, and 65% in high grade (Table 2). The frequency of CIP2A expression in high-grade tumor is significantly higher than that in low-grade tumor (*P* = 0.008). Our study also indicated that CIP2A protein expression levels increased concomitantly with the progression of bladder cancer. There were significantly more CIP2A-positive cases among patients with invasive disease (stage pT2 and above) compared with those with localized disease (stage pT1 and below) (Table 2, *P* = 0.00008). Only 12% of bladder cancer patients with pTa tumor overexpressed CIP2A protein. These data suggest that CIP2A protein expression was less frequent in low-risk and noninvasive tumors than high-risk and invasive tumors allowing this protein to serve as an indication of disease progression.

## Discussion

CIP2A, a recently characterized biomarker identified by Junttila et al., inhibits PP2A in human malignancies. CIP2A has been reported to be overexpressed in several human cancers, including head and neck squamous cell carcinoma, colon cancer, and gastric cancer. The CIP2A expression in bladder cancer has not been reported so far. Our study is the first to indicate an increased expression of CIP2A protein in human bladder TCC as compared with adjacent normal tissue.

For bladder cancer, the most reliable known prognostic markers are clinical and pathological stages of tumor, stage and grade. However, these markers are not sufficient to accurately predict the evolution and progression of invasive bladder cancers. CIP2A might serve as a prognostic marker because our study showed that overexpression of CIP2A protein was associated with tumorigenicity of bladder cancer and with high-grade, high-stage, invasive tumors. The protein expression frequency pattern of CIP2A is completely different from known protein biomarkers, such as survivin and DEK [17, 18]. For the same bladder cancer tissue panel, we also studied the protein expression of these two diagnostic markers, survivin and DEK, using Western blot assay (data not shown). We found that both survivin and DEK are present and overexpressed in over 80% of bladder cancer tissues regardless of clinical and pathological stage, which is consistent with previous literature report. Therefore, the CIP2A protein marker is unique among three proteins by strong association with the progression of the disease and by having the potential to predict the progression of disease while protein markers survivin and DEK cannot.

The tumorigenicity mechanisms of CIP2A protein also indicate it might have prognostic function in malignant disease. Chen et al. found that in hepatocellular carcinoma, CIP2A inhibited PP2A activity, upregulated phospho-Akt and Akt signaling pathway, and inhibited cell apoptosis [19, 20]. In cervical cancer, CIP2A functioned as an oncogene to regulate c-MYC degradation through inhibiting PP2A [8]. In gastric cancer, Zhao et al. showed that bacterial *Helicobacter pylori* oncogene CagA upregulated CIP2A expression and this upregulation effect was dependent on Src and MEK/ERK pathways [21, 22]. These pathways are known to play important roles in controlling the transformation and progression of bladder cancer. In addition, CIP2A protein has been reported as an independent predictor of poor prognosis in colon cancer [23]. CIP2A protein expression is also associated with poorly differentiated and high-risk prostate cancer [15]. These data suggest that CIP2A might be involved in the progression of bladder cancer and that the CIP2A might be a prognostic marker.

In summary, this preliminary study reports that CIP2A is an oncogene involved in bladder cancer as it is specifically expressed in bladder tumor tissue and not normal tissue. Also, the specific CIP2A protein expression frequency increased with increasing tumor grade and cancer stage, which suggests an association with the aggressiveness of bladder cancer. We understand that the prognostic function of CIP2A protein needs to be evaluated in a larger clinical study comparing CIP2A expression in primary and recurring tumors, as well as noninvasive and invasive tumors to determine the value of CIP2A protein detection as a prognostic tool. The overall sensitivity of CIP2A protein biomarker for TCC is about 42%. This result indicates that monitoring a single marker such as CIP2A does not have the sufficient sensitivity to be used for clinical diagnosis of bladder cancer. Therefore, it will be more powerful to identify a biomarker panel for clinical application. We also would like to emphasize that the combined sensitivity of CIP2A in noninvasive pTa and pT1 stages is only 19%, suggesting that CIP2A is not an ideal biomarker for the early detection of bladder cancer. Furthermore, the ability to measure CIP2A via a noninvasive method, such as urine analysis, might also be evaluated.
